# Iris-Claw Intraocular Lens Implantation in Various Clinical Indications: A 4-Year Study

**DOI:** 10.3390/jcm10061199

**Published:** 2021-03-13

**Authors:** Bogumiła Sędziak-Marcinek, Adam Wylęgała, Elżbieta Chełmecka, Mateusz Marcinek, Edward Wylęgała

**Affiliations:** 1 Chair and Department of Ophthalmology, Faculty of Medical Sciences in Zabrze, Medical University of Silesia, Panewnicka 65 Street, 40-760 Katowice, Poland; wylegala@gmail.com; 2 Health Promotion and Obesity Management Unit, Department of Pathophysiology, Faculty of Medical Sciences in Katowice, Medical University of Silesia, Medyków 65 Street, 40-728 Katowice, Poland; awylegala@sum.edu.pl; 3 Department of Statistics, Department of Instrumental Analysis, Faculty of Pharmaceutical Sciences, Medical University of Silesia, Ostrogórska 30, 41-200 Sosnowiec, Poland; echelmecka@sum.edu.pl; 4 Department of Urology, Faculty of Medical Sciences in Katowice, Medical University of Silesia, Plac Medyków 1, 41-200 Sosnowiec, Poland; matmarcinek@gmail.com

**Keywords:** aphakia, corneal transplants, endothelial cell count, iris-claw lens, intraocular pressure, postoperative complications, PPV, secondary IOL implantation, spherical equivalent

## Abstract

An iris-claw intraocular lens (IOL) has been widely used as a secondary implant in aphakic patients. The study presents the results of implanting the anterior chamber iris-claw Artisan IOL in cases of where an appropriate posterior capsular support is lacking. The study included 132 patients subjected to primary IOL implantation during complicated cataract surgery with damage to the posterior capsule (I), secondary implantation in aphakia (II), secondary implantation during penetrating keratoplasty (III), and secondary implantation during pars plana vitrectomy with luxated IOL extraction (IV). We analyzed the records of best-corrected visual acuity (BCVA), spherical equivalent (SE), intraocular pressure (IOP), and corneal endothelial cell count (cECC), taken before and 1, 2, 3, and 4 years after the surgery. BCVA depended on the time after IOL implantation and the primary indication. Four years post-surgery, the SE values were the lowest in group III. IOP was the same in all groups both before and after the surgery, but 4 years after the surgery IOP values in group IV were higher than in group III. The cECC decreased every year after the surgery in all groups, but four years after the IOL implantation, the lowest cECC values were observed in group IV. At the same time, all groups of patients showed improved BCVA, stable refraction, and a low percentage of postoperative complications.

## 1. Introduction

The lack of an appropriate posterior capsular support makes it impossible to implant the intraocular lens into the lens capsule or the ciliary sulcus. Aphakia and damage to the lens capsule most often occur as a complication after cataract surgery, an eye injury, aniridia, or the dislocation of a natural lens or an artificial intraocular lens (IOL) into the vitreous chamber [1,2]. The problem concerns about 1–3% of cataract surgeries, most often mature subluxation, traumatic and pseudoexfoliation cataracts [2]. Aphakia causes high hyperopia and anisometropia, significantly reducing the patient’s quality of life [3]. Alternative techniques for placing the intraocular lens in cases of posterior capsule damage include anterior chamber lens attached to the drainage angle, fixation with or without lining the sclera lens, anterior chamber or posterior iris lenses [4,5], and the use of a black diaphragm intraocular lens in aniridia [1].

An iris-claw anterior chamber lens (Artisan aphakic, Ophtec BV) was presented by Worst et al. in 1972, and since then it has been widely used in aperture correction [6]. It is a non-collapsible implant made of poly(methyl methacrylate) (PMMA), with an optical part of 5.4 mm diameter and a haptic part of 8.5 mm. The lens haptics are designed to be attached to the iris at a safe distance from the traverse angle and the corneal endothelium. In comparison to anterior chamber lenses, such a design reduces the risk of endothelial cells’ damage and the development of secondary glaucoma [7]. It also shortens and simplifies the procedure in comparison to attaching the lens to the sclera [8,9].

The analysis of the literature showed no studies describing the application of one type of lens in the different groups of patients. The study aimed to present the results of implanting an iris-claw lens in the anterior chamber in cases where an appropriate posterior capsular support was lacking, and compare the results for various groups of patients.

## 2. Materials and Methods

The study was conducted following the Declaration of Helsinki. Ethical approval was waived for this study, since it is a retrospective analysis of patients’ medical records only. All patients were informed about the details, goals, and risks of the operating procedure. Written consent for surgical intervention and for medical record analysis was obtained from all the patients before the study started.

The study evaluated best-corrected visual acuity, spherical equivalent, intraocular pressure and corneal endothelial cell count in patients that underwent iris-claw Artisan intraocular lens implantation. The parameters were analyzed at five time points of the study (before (time = 0) and 1, 2, 3 and 4 years after the IOL implantation surgery) for four different groups of patients, divided according to surgical indications for IOL implantation.

### 2.1. Study Inclusion Criteria

The study included patients qualified for the implantation procedure of an Artisan iris-fixated anterior chamber lens (Artisan Aphakia IOL model 205, Ophtec BV, Groningen, The Netherlands). The procedures were conducted in the Clinical Ophthalmology Department of the Regional Railway Hospital, Medical University of Silesia from 2012 to 2016. All the procedures were performed by the same surgeon.

The study included patients that matched the following inclusion criteria: age > 18, aphakia, absence of posterior capsular support, stable iris anatomy allowing IOL (intraocular lens) enclavation, anterior chamber depth > 3.00 mm, an endothelial cell density > 900/mm^2^ (except for patients with bullous keratopathy subjected to secondary iris-claw Artisan IOL implantation during penetrating keratoplasty), and a scheduled 4-year follow-up. Simultaneously, the study excluded patients with uncontrolled glaucoma with intraocular pressure (IOP) > 21 mmHg, retinal diseases, or history of uveitis. No patients who showed corneal opacities or any ocular co-morbidity that was judged to interfere with visual acuity improvement were included int the study. In addition, no patient included in the study wore contact lenses.

### 2.2. Diagnostic Methods

All patients enrolled in the study underwent a complete ophthalmological examination before the surgery and at every annual postoperative follow-up visit for four consecutive years. The ophthalmological examination included: slit lamp examination, best-corrected visual acuity (BCVA) test, refractive examination, intraocular pressure measurement, and corneal endothelial cell count (cECC) measurement. To analyze the changes in the corneal endothelial cell count in detail, the relative cECC was calculated (Equation (1)).

(1) $$cECC_{relative}=\frac{cECC_{after\;therapy}-cECC_{before\;therapy}}{cECC_{before\;therapy}}\;\times 100\%$$

All incidences of intra- and postoperative complications were also assessed.

BCVA was tested using the early treatment diabetic retinopathy study (ETDRS) chart testing method. The ETDRS charts 1 and 2 were printed with high-contrast lettering on a translucent white polystyrene panel. Chart 1 was placed 4 m from the patient in a back-illuminated stand, lit from behind, and displayed in a standard lightbox. The letters on the charts were arranged in 0.1 logMAR steps as specified in the ETDRS protocol (5 letters per line). The vision testing started with the first letter on the top row of the chart. If a patient could not read the largest letters at a normal distance, the chart was moved 50% closer to the patient (from 4 m to 2 m, or from 2 m to 1 m). The testing proceeded according to the forced-choice paradigm from the top of the chart to the bottom. It continued until the patient made a complete line of errors, read all letters on the chart, or could not read any letters of the chart when they were placed 1 m away from the patient. Patients were allowed to read the chart only one time, required to identify each letter, and encouraged to guess if not sure. The examiner pointed to each new line. The patients’ responses were marked on a scoring sheet with correctly identified letters circled on the sheet. The ETDRS chart was scored using a single letter scoring method with credit given for any letter correctly identified. If a patient failed to read any letters, they were assigned a 1.7 logMAR (Snellen equivalent 20/1000) score. The testing was repeated with chart 2 placed 2 m from a patient, using the same rules and scoring procedures [10].

The calculation of the iris-claw Artisan lens’ power was done with an IOLMaster^®^ 500 optical biometer (Carl Zeiss Meditec, Dublin, CA, USA) using the SRK-T formula. An A/B SCAN ultrasound (Quantel Medical, Cournon d’Auvergne, France) in the A projection was done when the eyeball’s distance from the optical biometer was reliable. The depth of the anterior chamber was also assessed with the IOLMaster^®^ 500 optical biometer. The intraocular pressure (IOP) was measured with a Goldman Applanation Tonometer (AT 900, Haag-Streit Diagnostics, Koniz, Switzerland). An SP-1P specular microscope (Topcon, Tokyo, Japan) with an autofocus function was used to calculate the corneal endothelial cell count (cECC). The cECC measurements were done automatically in the center of the cornea. Only one image was used for the calculations. Swept-source optical coherence tomography SS-OCT (DRI OCT Triton tomograph, Topcon, Japan) was used for the macular edema diagnosis and assessment at each visit during follow-up.

### 2.3. Study Groups

The study included 132 patients: 75 women (65%) and 57 men (45%). The average age of the patients was 70 (68–76) years (median (lower-upper quartile)). All patients underwent anterior chamber iris-claw Artisan IOL implantation. Depending on the clinical indications, the study distinguished four groups of patients:

- I—patients subjected to iris-claw Artisan IOL implantation during complicated cataract surgery with damage to the posterior capsule;
- II—patients subjected to secondary iris-claw Artisan IOL implantation in aphakia;
- III—patients with bullous keratopathy subjected to secondary iris-claw Artisan IOL implantation during penetrating keratoplasty;
- IV—patients subjected to secondary iris-claw Artisan IOL implantation during pars plana vitrectomy (PPV) with luxated IOL extraction.

The order of implants and number of eyes studied in each group of patients is presented in Table 1.

### 2.4. Operational Technique

An anterior chamber lens (type iris-claw) attached to the iris (Artisan Aphakia IOL model 205, Ophtec BV, Groningen, The Netherlands) was used as the IOL. The procedure was performed under peribulbar anesthesia.

**Figure 1:**
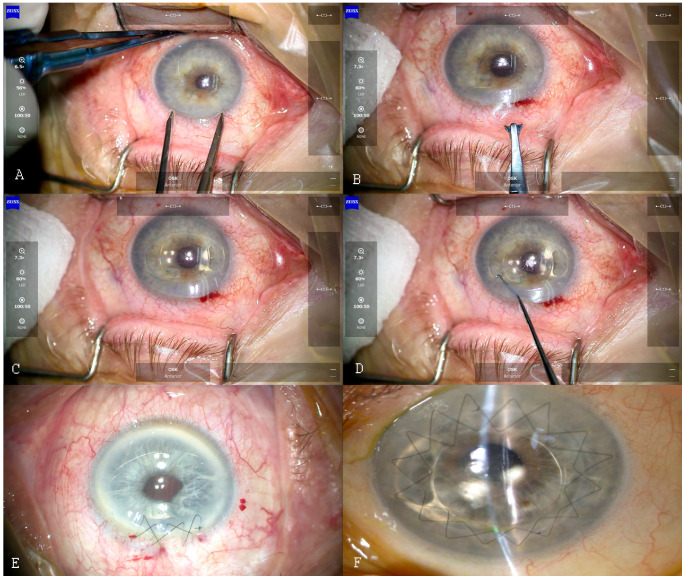
Iris-claw Artisan IOL (Artisan Aphakia IOL model 205, Ophtec BV) implantation technique: (**A**) paracenteses and corneal incision placement, (**B**) inserting the IOL into the anterior chamber, (**C**) centering the IOL over the pupil, (**D**) fixing the haptics to the iris mid-periphery using the enclavation needle, (**E**) suturing the main corneal incision, (**F**) implantation combined with penetrating keratoplasty. Please see the detailed description in the text.

Group I underwent IOL implantation during cataract phacoemulsification, complicated by damage to the posterior lens capsule. Group II underwent the secondary implantation in postoperative aphakia. The IOL implantation procedure was the same for patients from groups I, II and IV. Briefly, two 1.2 mm corneal vertical paracenteses directed towards the enclavation area, at the 10 and 2 o’clock positions, and one 5.2 mm corneal incision, centered at the 12 o’clock position, were performed (Figure 1A). After visualizing the vitreous body in the anterior chamber, the anterior vitrectomy was performed. Then, acetylcholine (1%) was applied to the anterior chamber to constrict the pupil, and dispersive and cohesive viscoelastic material was added to protect the corneal endothelium. The iris-claw Artisan IOL was inserted into the anterior chamber (Figure 1B), centered over the pupil with haptics at the 3 and 9 o’clock positions (Figure 1C). After checking the IOL position, the iris-claw haptics was fixed to the mid-periphery of the iris with an enclavation needle (Figure 1D). Next, a surgical peripheral iridectomy was performed at 12 o’clock. Finally, the corneal wound was sutured with 10–0 nylon non-absorbable sutures (Figure 1E). The tension of the sutures was checked with a standard Maloney keratoscope (Altomed, Boldon, UK). The viscoelastic material was removed with a bimanual irrigation/aspiration system (Infiniti™ Vision System; Alcon Laboratories, Inc., Fort Worth, TX, USA).

For the group of patients who underwent penetrating keratoplasty (III), the IOL was fixed to the iris, using the “open-sky” technique, after the recipient’s corneal tissue was excised with the keratotome. Attachment to the iris was performed using the same technique as above, followed by the corneal penetrating keratoplasty procedure with 10–0 non-absorbable nylon continuous double sutures (Figure 1F). In group IV patients, pars plana vitrectomy with removal of the artificial lens from the vitreous chamber was performed, followed by secondary lens implantation, as for the group I and II patients. In the study groups I, II, and IV, sutures were selectively removed starting from 5 months after the surgery. In group III, sutures were removed starting from 2 years after the surgery, depending on the topographic and refractive results. No subconjunctival steroids were given postoperatively.

### 2.5. Statistical Analysis

The distribution of variables was evaluated using the Shapiro–Wilk test and quantile–quantile plots. The interval data were expressed as a mean value ± standard deviation or median (lower-upper quartile) for skewed distribution. The four groups of patients were compared using the one-way ANOVA test (analysis of variance test) and the homogeneity of variance was verified by Levene’s test. The variables assessed several times during the study were analyzed using a two-way analysis of variance for repeated measures. The variation of the spherical assessment was based on Maucheley’s test. Statistical significance was set at *p* < 0.05 and all tests were two-tailed. Statistical analysis was performed using Statistica (data analysis software system) version 13.3 (TIBCO Software Inc., Palo Alto, CA, USA).

## 3. Results

The detailed results of the analyzed parameters for all study groups at all study time points are presented in Table S1 in the Supplementary Materials.

### 3.1. Best-Corrected Visual Acuity (BCVA)

**Figure 2:**
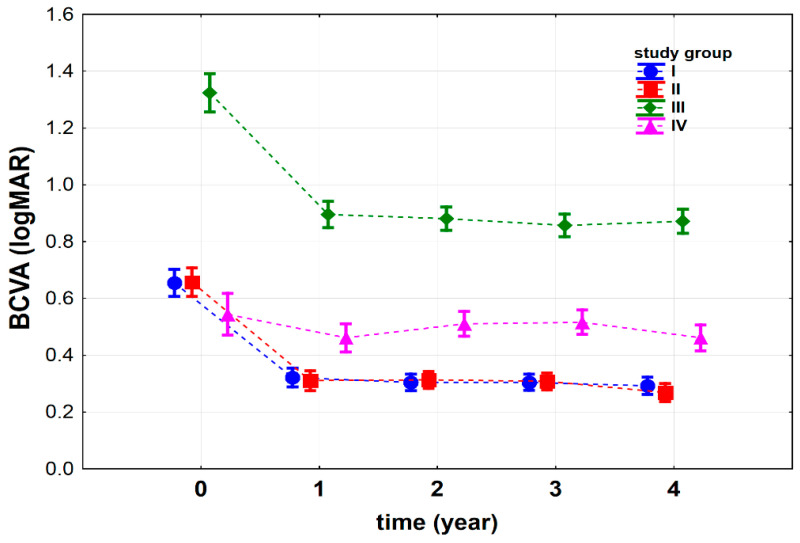
Best-corrected visual acuity (BCVA) (logMAR) before (t_0_) and after iris-claw Artisan intraocular lens (IOL) implantation in four groups of patients: I—patients subjected to complicated cataract surgery, II—patients with postoperative aphakia, III—patients with aphakia subjected to penetrating keratoplasty, IV—patients subjected to pars plana vitrectomy and luxated IOL extraction. Vertical lines represent 95% confidence intervals. For the reader’s convenience, the points relating to individual groups have been joined by a dashed line.

We observed that the best-corrected visual acuity (BCVA) significantly depended on the time after IOL implantation surgery (*p* < 0.001) and the interaction between the study groups (type of the surgery) and time (*p* < 0.001) (Figure 2). Before the surgery (time = 0), we found no statistical differences between groups I and II (*p* = 0.929). Group IV presented the lowest mean logMAR results of all the analyzed groups (p_I vs. IV_ < 0.05, p_II vs. IV_ < 0.05, and p_III vs. IV_ < 0.001). The highest logMAR results were observed for group III (p_I vs. III_ < 0.001, p_II vs. III_ < 0.001 and p_III vs. IV_ < 0.001). Similar results were obtained for 4 years after the surgery time point (p_I vs. II_ = 0.270, p_II vs. III_ < 0.001 and p_III vs. IV_ < 0.001) (Figure 2).

Considering each group separately, we observed a significant decrease in the logMAR results after the procedure in all groups (group I p_0 vs. 1_ < 0.001; group II p_0 vs. 1_ < 0.001; group III p_0 vs. 1_ <0.001; group IV p_0 vs. 1_ < 0.05), which indicated an improvement in their BCVA. Between the first and fourth years after the IOL implantation, we observed a plateau in the mean BCVA for groups I, II and III (group I: p_1 vs. 2_ = 0.359, p_1 vs. 3_ = 0.365, p_1 vs. 4_ = 0.136, p_2 vs. 3_ = 0.999, p_2 vs. 4_ = 0.515, p_3 vs. 4_ = 0.480; group II: p_1 vs. 2_ = 0.886, p_1 vs. 3_ = 0.892, p_1 vs. 4_ < 0.05, p_2 vs. 3_ = 0.739, p_2 vs. 4_ < 0.05, p_3 vs. 4_ < 0.05; group III: p_1 vs. 2_ = 0.562; p_1 vs. 3_ = 0.144; p_1 vs. 4_ = 0.376, p_2 vs. 3_ = 0.264, p_2 vs. 4_ = 0.712, p_3 vs. 4_ = 0.075). We noted that 4 years after IOL implantation, the patients from all the study groups showed lower logMAR results than before the surgery (for each group: p_0 vs. 4_ < 0.001) (Figure 2), which indicates that their BCVA improved after the IOL implantation procedure.

### 3.2. Refraction

**Figure 3:**
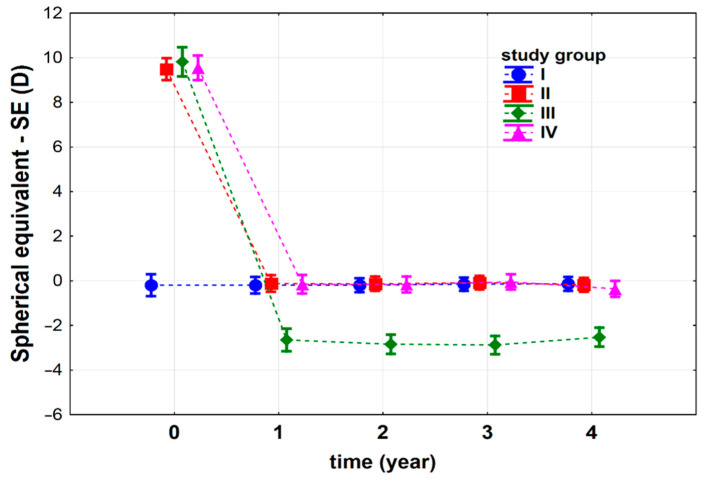
Spherical equivalent (SE) (D) before (t_0_) and after iris-claw Artisan intraocular lens (IOL) implantation in four groups of patients: I—patients subjected to complicated cataract surgery, II—patients with postoperative aphakia, III—patients with aphakia subjected to penetrating keratoplasty, IV—patients subjected to pars plana vitrectomy and luxated IOL extraction. The vertical lines represent the 95% confidence intervals. For the reader’s convenience, the points relating to individual groups are joined by a dashed line.

The analyses of the spherical equivalent (SE) measurements showed that refraction also depended on the time after the IOL implantation surgery (*p* < 0.001), and the interaction between the study group (type of the surgery) and the time (*p* < 0.001). We observed that the SE for group I before the surgery (time = 0) was significantly lower than for other groups (p_I vs. II_ < 0.001, p_I vs. III_ < 0.001, and p_I vs. IV_ < 0.001), while the results for the other groups did not differ (p_II vs. III_ = 0.435, p_II vs. IV_ = 0.873, and p_III vs. IV_ = 0.542) (Figure 3). Such results were expected as the patients from groups II–IV were aphakic and required approximately 10 dioptres of ocular correction for visual acuity improvement. Therefore, the SE values for these groups are similar, but are higher in comparison to group I.

Comparing the results of the refraction measurements 4 years after the surgery, we found that the SE values of patients from group III were the lowest, while the SE values of the patients from groups I, II and IV were higher, and did not differ from each other (p_I vs. II_ = 0.828, p_I vs. III_ < 0.001, and p_I vs. IV_ = 0.357; p_II vs. III_ < 0.001 and p_II vs. IV_ = 0.472, and finally p_III vs. IV_ < 0.001) (Figure 3). This may indicate that refraction improvement is less predictable after corneal transplantation than after the other types of procedures performed during the IOL implantation.

Considering each group separately, we found that in group I, the IOL implantation procedure did not change the SE value (p_0 vs. 1_ = 0.996, p_0 vs. 2_ = 0.992, p_0 vs. 3_ = 0.870, p_0 vs. 4_ = 0.835). In groups II, III and IV, the SE value decreased significantly after surgery (group II p_0 vs. 1_ < 0.001, group III p_0 vs. 1_ < 0.001, group IV p_0 vs. 1_ < 0.001) and reached a plateau between the first and fourth years after the surgery (Figure 3). The results indicate that the performed IOL implantation procedures were successful in all groups of patients, and the refraction was stable over the 4-year observation period.

### 3.3. Intraocular Pressure (IOP)

We observed that the intraocular pressure depended only on the study group (type of the surgery) (p_group_ < 0.001, p_time_ = 0.118, p_interaction_ = 0.504). We analyzed in detail the IOP results for individual groups, and we found that the IOP was the same for all study groups before (p_I vs. II_ = 0.083, p_I vs. III_ = 0.068, p_I vs. IV_ = 0.800, p_II vs. III_ = 0.693, p_II vs. IV_ = 0.066, p_III vs. IV_ = 0.054) and 4 years after the IOL implantation procedure (p_I vs. II_ = 0.480, p_I vs. III_ = 0.110, p_I vs. IV_ = 0.337, p_II vs. III_ = 0.315, p_II vs. IV_ = 0.114), with only one exception for groups III and IV. We observed that 4 years after the IOL implantation procedure IOP values of group IV were significantly higher when compared to group III (17.0 ± 2.9 vs. 15.4 ± 1.4 mmHg, respectively; p_III vs. IV_ < 0.05) (Table S1).

### 3.4. Corneal Endothelial Cell Count (cECC)

**Figure 4:**
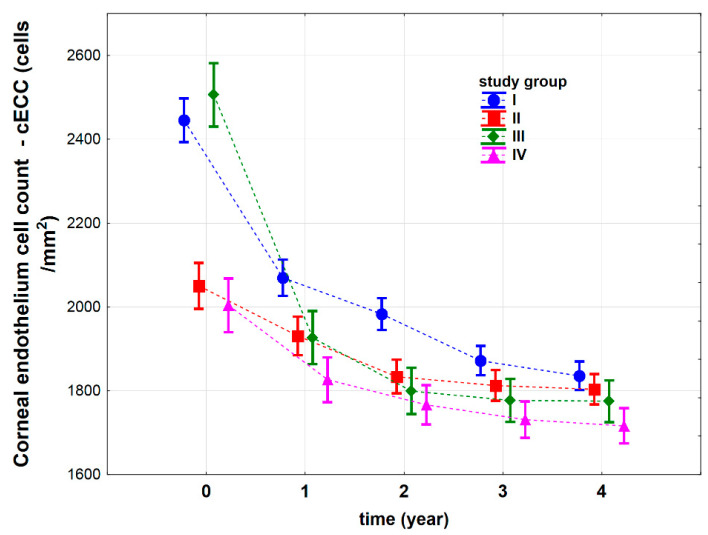
Corneal endothelium cell count (cECC) (cells/mm^2^) before (t_0_) and after iris-claw Artisan intraocular lens (IOL) implantation in four groups of patients: I—patients subjected to complicated cataract surgery, II—patients with postoperative aphakia, III—patients with aphakia subjected to penetrating keratoplasty, IV—patients subjected to pars plana vitrectomy and luxated IOL extraction. Vertical lines represent 95% confidence intervals. For the reader’s convenience, the points relating to individual groups have been joined by a dashed line.

We found that corneal endothelial cell count depended on time after the IOL implantation procedure (*p* < 0.001) and an interaction between the study group (type of the surgery) and time (*p* < 0.001). We found no significant differences between the cECC values for groups I and III, or between the cECC values for groups II and IV, before the surgery (p_I vs. III_ = 0.195, p_II vs. IV_ = 0.281) (Figure 4). The cECC values for groups I and II were significantly higher than those for groups II and IV (p_I vs. II_ < 0.001, p_I vs. IV_ < 0.001, p_II vs. III_ < 0.001, and p_III vs. IV_ < 0.001). The higher cECC values for group I result from the fact that patients from this group did not undergo any previous surgery, which usually damages the cornea and decreases cECC. Additionally, the higher cECC value for group III may result from the fact that it was measured for the donor flap. Groups II and IV were previously subjected to cataract surgery, hence the lower initial cECC values for these groups, which was expected. Four years after the IOL implantation procedure, the lowest cECC values were observed in group IV (p_I vs. II_ = 0.200, p_I vs. III_ < 0.05, p_I vs. IV_ < 0.001, p_II vs. III_ = 0.363, p_II vs. IV_ < 0.01, p_III vs. IV_ = 0.080), which suggests that pars plana vitrectomy is the most invasive procedure.

Individual analysis of corneal endothelial cell count for each group showed a systematic decrease in cECC values every year after the IOL implantation procedure (group I p_0 vs. 4_ < 0.001, group II p_0 vs. 4_ < 0.001, group III p_0 vs. 4_ < 0.001, group IV p_0 vs. 4_ < 0.001) (Figure 4).

**Figure 5:**
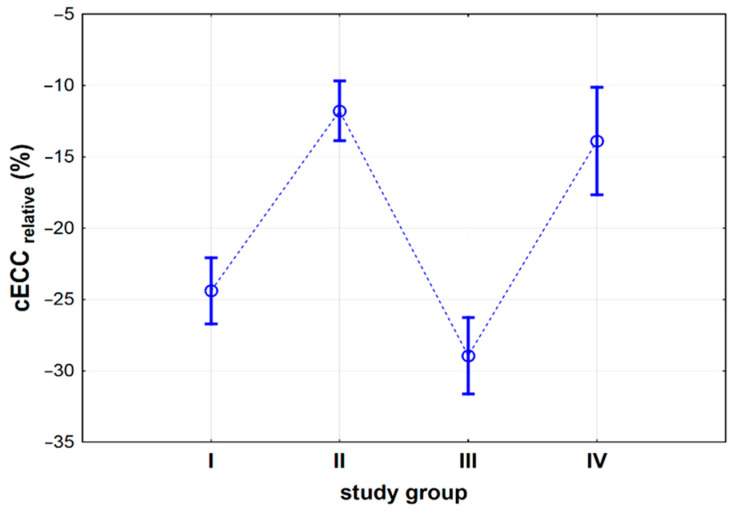
Relative corneal endothelium cell count (cECC_relative_) (%) in four groups of patients subjected to iris-claw Artisan intraocular lens (IOL) implantation: I—patients subjected to complicated cataract surgery, II—patients with postoperative aphakia, III—patients with aphakia subjected to penetrating keratoplasty, IV—patients subjected to pars plana vitrectomy and luxated IOL extraction. Vertical lines represent 95% confidence intervals. For the convenience of the reader, the dots are connected by a dashed line.

We found that cECC_relative_ depended on the type of the surgery performed (study group) in patients subjected to an IOL implantation procedure (*p* < 0.001, Table S2). We observed no significant differences in cECC_relative_, calculated individually, for groups I and III (p_I vs. III_ = 0.217) and groups II and IV (p_II vs. IV_ = 0.711). Groups I and III showed greater percentage changes in their cECC_relative_ (p_I vs. II_ < 0.001, p_I vs. IV_ < 0.001, p_II vs. III_ < 0.001, p_III vs. IV_ < 0.001) (Figure 5).

Additionally, the analysis showed that the cECC parameter changed every year and the changes were statistically significant (*p* < 0.001) (Figure S1). The greatest loss in cECC, of 280 (115–438) cells/mm^2^ (median (lower-upper quartile)), was observed in the first year after the implantation procedure. In the second year, we observed a cECC loss of 70 (20–125) cells/mm^2^ on average, when compared to the first year. In the third year, the cECC was reduced by 40 (10–100) cells/mm^2^ when compared to the second year. In the fourth year, when compared to the third year, we also observed a reduction in the cECC parameter by 20 (-10–50) cells/mm^2^. Additionally, in the fourth year after the IOL implantation, 34% of patients expressed no change, or even showed an increase in the cECC parameter when compared to the previous year (this explains the negative number for the lower quartile).

### 3.5. Intra- and Postoperative Complications

**Figure 6:**
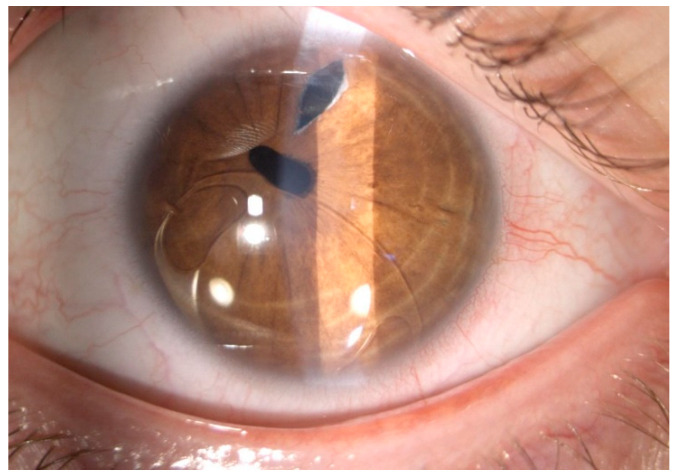
Slit-lamp photograph of a subluxated anterior chamber iris-claw (Artisan) IOL.

The analysis of the operative protocols showed no intraoperative complications. Postoperative complications were rare, and included 4.54% of IOL subluxation (*n* = 6) (Figure 6), 3.03% of cystoid macular edema (*n* = 4), and 1.51% of retinal detachment (*n* = 2).

## 4. Discussion

To our knowledge, the presented study is the most extensive case series of anterior chamber Artisan iris-claw IOL implantation procedures, analyzed in various groups of patients, including those after penetrating keratoplasty or pars plana vitrectomy (PPV). Four years after the IOL implantation procedure, all groups of patients showed improved best-corrected visual acuity (BCVA), stable refraction, and a low percentage of postoperative complications. We showed that aphakia correction with anterior chamber iris-claw Artisan IOL is applicable in various groups of patients.

The BCVA significantly improved in all groups of patients enrolled in our study. Our results for the BCVA, and the significant reduction in the spherical equivalent observed in patients with aphakia and bullous keratopathy, agree with results obtained by Kanellopoulos [11]. In patients after PPV with luxated IOL extraction (group IV), we observed a lower improvement in BCVA than in other study groups. The BCVA improvement observed in group IV was similar to the results of Labeille et al. [12]. They found that iris-claw IOL implantation in aphakia after PPV with luxated IOL extraction is both effective and safe. Their study showed that after the surgery, patients presented a stable spherical equivalent and a significant improvement in visual acuity [12].

Currently, the greatest problem for patients with a ruptured capsular bag and requiring IOL implantation is related to postoperative complications. Postoperative complications often include damage to the corneal endothelial cells, corneal decompensation, secondary glaucoma, secondary lens dislocation, macular edema, hemorrhagic complications, and retinal detachment [9]. Wagoner et al. reported that complications depend on the type of IOL applied as a secondary implant, and bullous keratopathy is the most frequent complication for angle-supported IOL [9]. They also reported that scleral-fixated IOL increases the risk of intravitreal or suprachoroidal hemorrhage during the procedure, and scleral fixation sutures increase the risk of endophthalmitis [13]. Improper alignment between IOL and sclera causes the IOL to shift and the sutures to rupture, which dislocates the lens into the vitreous chamber [13]. Lens dislocation and the detachment of the haptic attached to the iris are other frequent complications of iris-claw IOL implantation that have been reported in the literature [14,15]. In the case of the iris-claw IOL attached to the posterior surface of the iris, detachment of the haptics or luxation of the IOL in the course of severe eye trauma results in the dislocation of the IOL into the posterior chamber. De Silva et al. showed that 6% of all iatrogenic IOL displacements occurred within 5–60 days after surgery, as a result of rigid haptics in older models of lenses [4]. In our study, 4.54% of patients experienced lens subluxation in the anterior chamber caused by haptic release due to an eye injury, and all of them underwent haptic reenclavation to the iris without intraoperative and postoperative complications. Similarly, other studies reported that re-fixation of the haptic to the iris is not a complicated procedure [16,17]. Although some authors advocate the use of light diathermy when the iris’ shape is distorted [18], we did not use that technique.

Additionally, we observed no case of a decompensation of the corneal graft or a secondary increase in intraocular pressure during the 4 years of the study. Kanellopoulos et al. also observed, at a 24-month follow-up, that all corneal grafts remained clear [11]. Gonnermann et al. observed no signs of decompensation of the corneal graft in patients after posterior iris-claw lens up to 18 months after the procedure [19]. As for the intraocular pressure, the literature reports that it concerns only 3–5% of incidences [14,16]. Our observations on the safe and efficient use of iris-claw anterior chamber IOL (artisan type) agree with other reports [14,20,21]. Moreover, its implantation procedure is easier and shorter than other surgical techniques, such as the scleral fixation of the posterior chamber iris-claw lens. This type of procedure also reduces the risk of postoperative macular edema, intraocular hemorrhagic complications, and retinal detachments [15,22]. Vote et al. [23] and Bading et al. [24] reported, respectively, 6.3–8.2% of retinal detachment, and 3.2% of choroidal hemorrhage incidence, after the posterior ventricular lens implant with scleral fixation. In our study, we observed 1.51% of retinal detachment and 3.03% of postoperative macular edema incidence for all study patients.

Despite the technological progress, the currently used methods of secondary implant application are associated with some risks of complications. Among them, the iris-attached IOL implantation has the lowest number of postoperative complications and the earliest sound postoperative effects [9]. Shuaib et al. compared the sutureless transscleral intraocular lens fixation to retropupillary iris-claw lens implantation in children with aphakia without capsular support. Their results showed a comparable visual outcome and a similar loss of cECC in both groups of patients (11% for transscleral IOL and 14.6% for iris-claw IOL) 6 months after the surgery [25]. Frisina et al. described that needle-guided retropupillary iris-claw IOL (RP-ICIOL) was easier to perform than the scleral-fixation technique. RP-ICIOL reduced the surgical manipulations of the IOL into the anterior chamber, and facilitated the enclavation and accuracy of the implantation [26]. The loss of corneal endothelial cells after iris-fixation is a widely studied complication. Güell et al. found a statistically significant (5.94%) decrease in cECC during the first 5 years after secondary iris-claw Artisan IOL (Ophtec BV) implantation [14]. In other work, Güell et al. observed the highest reduction in endothelial cell density one year after the Artisan–Verysise IOL secondary implantation for aphakia correction surgery [27]. In our study, we observed a different decrease in cECC_relative_ during complicated cataract surgery when comparing the study groups with secondary and primary implants, which agrees with other studies [14,28]. In all studied groups, we observed reduced cECC after the surgery, with the greatest decrease in the number of endothelial cells for patients from group I (subjected to complicated cataract surgery with damage to the posterior capsule and primary IOL implantation) and group III (subjected to keratoplasty with a penetrating corneal graft and secondary IOL implantation). The loss of corneal endothelial cells may also affect patients that chronically use contact lenses instead of having the secondary IOL implanted, as observed by Pallikaris et al. [29]. Toro et al. in a 5-year study, showed that using IOL attached to the iris anterior chamber did not affect cECC when compared to posterior chamber lenses attached to the posterior surface of the iris [18]. In addition, Mora et al. compared the functional and clinical outcomes of the iris-claw IOL placed on the anterior and posterior surfaces of the iris. They showed that fixing the iris-claw IOL on the anterior or posterior chamber is equally effective and safe for aphakic eyes with inadequate capsular support [30]. Other reports showed no changes in cECC after iris-fixed lens implantation when compared with sulcus-fixated lens implantation [8].

On the other hand, Teng and Zhang described a moderate loss of corneal endothelial cells after the use of an iris-attached anterior chamber lens, but this was comparable to the sulcus fixation lens [22]. It seems that one of the most critical factors for cECC changes is time elapsed after surgery, and the close monitoring of the patients [14]. Sminia et al. investigated the effect of the anterior chamber iris-attached lenses on the density of corneal endothelial cells in children for almost 10 years. They found no differences in the density of corneal endothelial cells between the studied group and the population [31].

In our study, patients from groups II and IV initially underwent cataract surgery, which reduced the number of endothelial cells. Therefore, before the second surgery of secondary IOL implantation, at the beginning of this study, we observed initially lower cECC values in these groups. Patients from group I did not undergo any surgery before the beginning of this study, hence the higher initial number of corneal endothelial cells noted for this group of patients. In group III, the endothelial cell density measurement included the corneal graft surface, which resulted in initial high cECC values. The decrease in cECC values noted for group II patients 4 years after the surgery was comparable to the results of Gonnermann et al. [19] for patients 18 months after undergoing an iris-claw lens attachment to the rear chamber IOL implantation procedure. We conclude that the anterior chamber iris-claw Artisan IOL implantation reduces the number of corneal endothelial cells, but no more so than standard cataract surgery. The changes in cECC depend on the type of surgery performed during IOL implantation.

The major limitation of our study is the lack of a control group and the restricted number of patients in the consecutive study groups. However, this paper provides data on the primary vs. secondary implantation of an Artisan IOL. Due to the different techniques used for IOL implantation and the different indications for the procedure, a comparison between phacoemulsification with IOL implantation and iris-claw IOL implantation would not be justified. The second limitation of the presented paper is the cECC measurement being based only on one image.

## 5. Conclusions

Using an anterior chamber lens fixed to the iris as an intraocular lens implantation method seems to be an effective and safe technique for aphakia correction in various groups of patients with posterior lens capsule damage. Iris-claw anterior chamber IOL implantation presents a low risk of complications, and induces a cECC loss that depends on the type of surgical procedure performed. The postoperative visual acuity prognosis for this type of IOL depends on the primary diagnosis of the patient.

## Figures and Tables

**Table 1:** Groups of patients subjected to iris-claw Artisan intraocular lens (IOL) implantation.

Study Group	Surgical Indication	Implant Order	Number of Eyes
I	cataract surgery with damage to the posterior capsule	primary	42
II	postoperative aphakia	secondary	39
III	aphakia with bullous keratopathy	secondary	21
IV	PPV and luxated IOL extraction	secondary	30

Abbreviations: IOL—intraocular lens, PPV—pars plana vitrectomy.

## Data Availability

The data presented in this study are available on request from the corresponding author.

## Supplementary Materials

The following are available online at https://www.mdpi.com/2077-0383/10/6/1199/s1, Table S1. Best-corrected visual acuity (BCVA), spherical equivalent (SE), intraocular pressure (IOP), and corneal endothelium cell count (cECC) measured before (baseline) and 1, 2, 3 and 4 years after after iris-claw. Artisan intra-ocular lens (IOL) implantation in four groups of patients: I—patients subjected to complicated cataract surgery, II–patients with postoperative aphakia, III–patients with aphakia subjected to penetrating keratoplasty, IV–patients subjected to vitrectomy and luxated IOL extraction. Table S2. Age and relative corneal endothelium cell count (cECCrelative) of patients subjected to iris-claw Artisan in-traocular lens (IOL) implantation from different study groups: I—patients subjected to complicated cataract sur-gery, II—patients with postoperative aphakia, III—patients with aphakia subjected to penetrating keratoplasty, IV–patients subjected to vitrectomy and luxated IOL extraction. Figure S1. Yearly change in the corneal endothelial cell count (cECC) [cells/mm2] in patients subjected to iris-claw Artisan intraocular lens (IOL) implantation during a 4-year postoperative period.
