# Supplementary material for: Iris-Claw Intraocular Lens Implantation in Various Clinical Indications: A 4-Year Study
Source: J Clin Med. 2021 Mar 13;10(6):1199. doi: 10.3390/jcm10061199 (PMC7998776; doi:10.3390/jcm10061199)

Supplementary Materials

Table S1. Best-corrected visual acuity (BCVA), spherical equivalent (SE), intraocular pressure (IOP) , and corneal endothelium cell count (cECC) measured before (baseline) and 1, 2, 3 and 4 years after after iris-claw. Artisan intraocular lens (IOL) implantation in four groups of patients: I—patients subjected to complicated cataract surgery, II—patients with postoperative aphakia, III—patients with aphakia subjected to penetrating keratoplasty, IV—patients subjected to vitrectomy and luxated IOL extraction. Results are presented as mean and standard deviation. The results were analyzed using a ANOVA test (analysis of variance test). Statistical significance was set at $p < 0.05$.

Analyzed parameters	Study group	Baseline	1 year	2 years	3 years	4 years	p_{ANOVA}
BCVA [logMAR]	I ($n = 42$)	0.7 ± 0.1	0.3 ± 0.1	0.3 ± 0.1	0.3 ± 0.1	0.3 ± 0.1	< 0.001
	II ($n = 38$)	0.7 ± 0.1	0.3 ± 0.1	0.3 ± 0.1	0.3 ± 0.1	0.3 ± 0.1	
	III ($n = 21$)	1.3 ± 0.3	0.9 ± 0.1	0.9 ± 0.1	0.9 ± 0.1	0.9 ± 0.1	
	IV ($n = 18$)	0.5 ± 0.1	0.4 ± 0.1	0.5 ± 0.1	0.5 ± 0.1	0.5 ± 0.1	
SE [D]	I ($n = 37$)	-0.20 ± 1.44	-0.23 ± 1.04	-0.22 ± 0.90	-0.19 ± 0.69	-0.17 ± 0.66	< 0.001
	II ($n = 37$)	9.44 ± 1.42	-0.12 ± 0.71	-0.09 ± 0.73	-0.04 ± 0.71	-0.16 ± 0.70	
	III ($n = 20$)	9.69 ± 1.65	-2.63 ± 1.68	-2.80 ± 1.28	-2.80 ± 1.33	-2.52 ± 1.42	
	IV ($n = 29$)	9.59 ± 1.57	-0.15 ± 1.16	-0.20 ± 1.02	-0.03 ± 1.07	-0.38 ± 1.14	
IOP [mmHg]	I ($n = 42$)	16.4 ± 3.9	16.1 ± 2.7	16.1 ± 2.5	16.4 ± 2.1	16.5 ± 2.5	< 0.001
	II ($n = 38$)	15.2 ± 2.2	16.1 ± 2.4	15.3 ± 2.1	15.9 ± 2.0	16.1 ± 2.6	
	III ($n = 20$)	15.0 ± 1.5	15.1 ± 1.1	15.0 ± 1.2	14.8 ± 1.2	15.4 ± 1.4	
	IV ($n = 30$)	16.6 ± 3.5	17.9 ± 2.8	17.1 ± 2.7	16.6 ± 1.6	17.0 ± 2.9	
cECC [cells/mm^2]	I ($n = 42$)	2446 ± 219	2070 ± 145	1983 ± 124	1872 ± 119	1836 ± 106	< 0.001
	II ($n = 38$)	2050 ± 126	1932 ± 108	1836 ± 113	1815 ± 111	1805 ± 99	
	III ($n = 20$)	2524 ± 192	1926 ± 206	1799 ± 122	1777 ± 104	1775 ± 117	
	IV ($n = 28$)	2008 ± 130	1822 ± 127	1765 ± 144	1728 ± 119	1716 ± 135	

Legend: I—patients subjected to complicated cataract surgery, II—patients with postoperative aphakia, III—patients with aphakia subjected to penetrating keratoplasty, IV—patients subjected to vitrectomy and luxated IOL extraction, BCVA–best-corrected visual acuity cECC—corneal endothelium cell count , IOP—intraocular pressure, SE—spherical equivalent.

Table S2. Age and relative corneal endothelium cell count ($cECC_{relative}$) of patients subjected to iris-claw Artisan intraocular lens (IOL) implantation from different study groups: I—patients subjected to complicated cataract surgery, II—patients with postoperative aphakia, III—patients with aphakia subjected to penetrating keratoplasty, IV—patients subjected to vitrectomy and luxated IOL extraction. Results are presented as mean standard deviation. The results were analyzed using a one-way ANOVA test (analysis of variance test). Statistical significance was set at $p < 0.05$.

	**Study group**				
	**I ($n = 42$)**	**II ($n = 39$)**	**III ($n = 21$)**	**IV ($n = 30$)**	p_{ANOVA}
Age [years]	70.3 ± 5.7	69.7 ± 6.8	73.0 ± 4.6	69.7 ± 6.2	0.182
$cECC_{relative}$ [%]	-24 ± 7	-12 ± 6	-29 ± 6	-14 ± 10	< 0.001

Legend: I—patients subjected to complicated cataract surgery, II—patients with postoperative aphakia, III—patients with aphakia subjected to penetrating keratoplasty, IV—patients subjected to vitrectomy and luxated IOL extraction, $cECC_{relative}$—relative corneal endothelium cell count.

Figure S1. Yearly change in the corneal endothelial cell count (cECC) [cells/mm²] in patients subjected to iris-claw Artisan intraocular lens (IOL) implantation during a 4-year postoperative period.

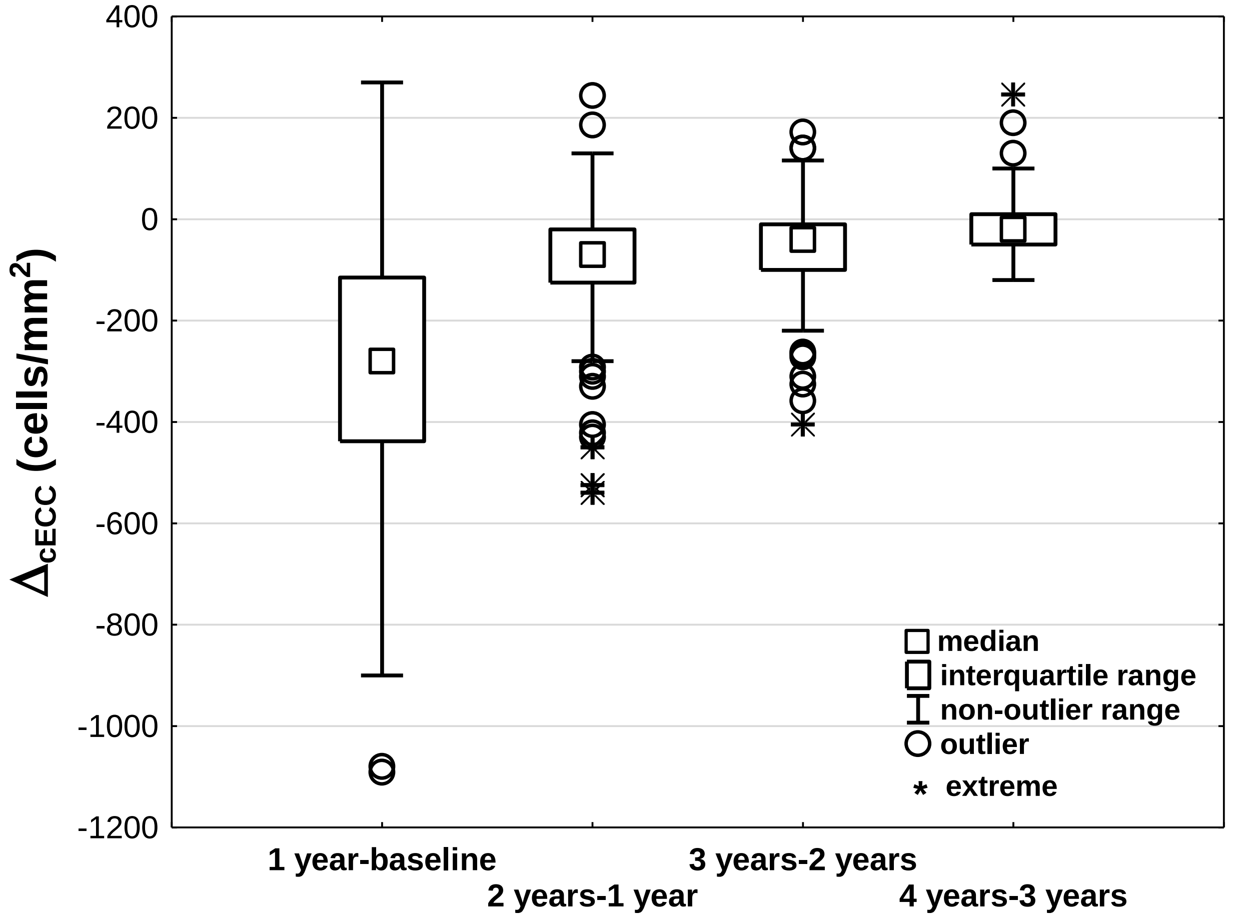

Supplement: Supplementary file 1 [file jcm-10-01199-s001.pdf]
